# Characterisation of *Anopheles* strains used for laboratory screening of new vector control products

**DOI:** 10.1186/s13071-019-3774-3

**Published:** 2019-11-05

**Authors:** Jessica Williams, Lori Flood, Giorgio Praulins, Victoria A. Ingham, John Morgan, Rosemary Susan Lees, Hilary Ranson

**Affiliations:** Department of Vector Biology, Liverpool School of Tropical Medicine, Liverpool, UK

**Keywords:** Vector control, Insecticide, *Anopheles*, Malaria, Product screening, Resistance phenotypes, Resistance genotypes

## Abstract

**Background:**

Insecticides formulated into products that target *Anopheles* mosquitos have had an immense impact on reducing malaria cases in Africa. However, resistance to currently used insecticides is spreading rapidly and there is an urgent need for alternative public health insecticides. Potential new insecticides must be screened against a range of characterized mosquito strains to identify potential resistance liabilities. The Liverpool School of Tropical Medicine maintains three susceptible and four resistant *Anopheles* strains that are widely used for screening for new insecticides. The properties of these strains are described in this paper.

**Methods:**

WHO tube susceptibility bioassays were used for colony selection and to screen for resistance to the major classes of public health insecticides. Topical and tarsal contact bioassays were used to produce dose response curves to assess resistance intensity. Bioassays with the synergist piperonyl butoxide were also performed. Taqman™ assays were used to screen for known target site resistance alleles (*kdr* and *ace-1*). RT-qPCR was used to quantify expression of genes associated with pyrethroid resistance.

**Results:**

Pyrethroid selection pressure has maintained resistance to this class in all four resistant strains. Some carbamate and organophosphate resistance has been lost through lack of exposure to these insecticide classes. The *Anopheles gambiae* (*sensu lato*) strains, VK7 2014, Banfora M and Tiassalé 13 have higher levels of pyrethroid resistance than the *An. funestus* FUMOZ-R strain. Elevated expression of P450s is found in all four strains and the 1014F *kdr* mutation is present in all three *An. gambiae* strains at varying frequencies. Tarsal contact data and overexpression of *CYP4G16* and *SAP2* suggest penetration barriers and/or sequestration also confer resistance in Banfora M.

**Conclusions:**

Continual selection with deltamethrin has maintained a stable pyrethroid-resistant phenotype over many generations. In conjunction with a standardized rearing regime, this ensures quality control of strains over time allowing for robust product comparison and selection of optimal products for further development. The identification of multiple mechanisms underpinning insecticide resistance highlights the importance of screening new compounds against a range of mosquito strains.
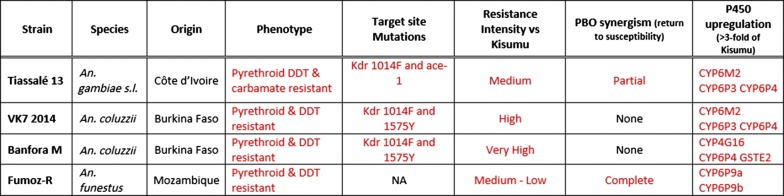

## Background

Insecticides play a pivotal role in malaria control. The scale-up in use of long-lasting insecticidal nets (LLINs) and, to a lesser extent, indoor residual spraying (IRS), has had an immense impact in Africa, where malaria cases have halved since the beginning of the century [1]. However, the intense selection pressure on malaria mosquitoes has led to insecticide resistance, which is decreasing the impact of vector control in some settings [1–3]. Indeed, over the past two years, progress against malaria has stalled [4] highlighting the need for new chemicals or other tools and strategies that can control insecticide-resistant vectors.

Until 2019, pyrethroids were the only insecticide class used in LLINs, with some of the LLINs deployed since 2017 also containing the insecticide synergist, piperonyl butoxide (PBO) to increase their efficacy against resistant mosquitoes [5]. Only four additional classes of insecticides are currently used for IRS: organophosphates, carbamates, organochlorines and, recently, neonicotinoids [4]. Resistance to all these insecticide classes, with the exception of neonicotinoids, has been reported in African malaria vectors with pyrethroid resistance particularly prevalent [4]. Pyrethroid resistance is conferred by two well-characterised mechanisms, modifications in the target site, the voltage gated sodium channel (known as *kdr* alleles) and elevated rates of insecticide detoxification typically caused by overexpression of cytochrome P450 genes [6, 7]. More recently, modifications in the insect cuticle, reducing insecticide penetration [8] and elevated expression of putative pyrethroid-binding proteins [9, 10] have also been implicated in pyrethroid resistance in *An. gambiae* (*s.l.*).

The Innovative Vector Control Consortium (IVCC) [11] was established in 2005 as a not-for-profit, product development partnership (PDP) to facilitate the development and delivery of new and improved vector control tools to prevent malaria and other neglected tropical diseases. One of the key workstreams of this PDP is to work with the major agrochemical companies to identify, develop and evaluate new lead chemistries for use in vector control tools. A critical step in this process is the screening of these new insecticide chemistries for efficacy against a range of mosquito populations to identify any cross-resistance risks at an early stage in the product development pipeline [12]. To facilitate this, in 2011, the Liverpool School of Tropical Medicine (LSTM), established a unit dedicated to the testing of insecticide-based products known as the Liverpool Insect Testing Establishment (LITE). LITE provides a service to industrial partners to screen new and repurposed insecticides using a range of standard and bespoke bioassays. Examples of the activities of LITE include the screening of existing insecticide classes to identify those that had the potential to be re-purposed for use in public health [12], screening of novel active ingredients supplied by industry partners, and assessing the durability of various insecticide formulations on different surfaces.

LITE maintains a range of insecticide susceptible and resistant mosquito species and strains representing key known resistance mechanisms. Strict quality standards are employed within LITE for rearing and testing to improve the consistency of results and allow for comparison between compounds. To retain a stable resistance phenotype, strains are maintained under insecticide selection pressure and they are routinely monitored using a series of phenotypic bioassays and genotyping methodologies. Upon arrival into LITE all strains are initially screened for three mutations in the voltage-gated sodium channel (L1014F, L1014S and N1575Y *kdr* mutations) the target site of pyrethroids and DDT, and one acetylcholine esterase mutation (G119S known as *ace*-1), the target site for organophosphates and carbamates; if detected, their frequency is monitored on a regular basis. These phenotypic and genotypic characterizations are performed to identify the resistance mechanisms carried by each strain, to ensure that the strains maintained in LITE represent all major resistance mechanisms found in the field in a ‘suite’ of resistant strains to screen for cross-resistance.

Establishing and maintaining multiple different mosquito populations comes with many challenges, including adapting field populations to a laboratory environment, maintaining stable resistance phenotypes, quantifying resistance levels, and deciphering major resistance mechanisms. Here, we describe the resistance phenotypes of seven *Anopheles* strains maintained by LITE including a description of the rearing and selection schedule with information on the stability of resistance over multiple generations. We also describe the range of bioassays and genotyping assays that are routinely used to screen these strains and present data on the stability of these traits over time. This information will be of interest to innovators wishing to evaluate the performance of potential new vector control products against a range of insecticide-resistant populations and will, we hope, also aid other groups in establishing, maintaining and characterising stable populations of insecticide resistant mosquitoes in insectaries.

## Methods

### Establishment of strains

Details of the origin of the strains are provided in Table 1. For all field collections, blood-fed females were isolated in Eppendorf tubes for ‘forced egg-layingʼ [13]. Females that laid eggs were then separated and stored on silica. The tubes with eggs were then brought to Liverpool under licence. Each isofemale egg batch was hatched in a paper cup containing purified (Millipore) water and larvae fed on ground fish food (Tetramin Tropical Flakes). PCR [14] was performed on the female parent and 6 individual larvae from each cup to identify members of the same species within a morphologically identical species complex. *Anopheles gambiae* (*s.s.*) or *Anopheles coluzzii* isofemale lines were then either pooled by species, or discarded, to establish a single strain of the predominant species (Table 1). These field strains were initially provided blood meals from a volunteer’s forearm, before being transferred to feeding on artificial membranes (see ‘Mosquito rearing’). Adaptation to membrane feeding can take many generations, and parallel strains were maintained on arm feeding to safeguard the colony during this process.

**Table 1:** Strains origin information

Strain name	Species	Origin	Source	Year colony established at LSTM
Kisumu	*An. gambiae* (*s.s.*)	Kenya	MR4^a^	1975
Moz	*An. arabiensis*	Mozambique	Established in LSTM from field collections [38]	2009
Tiassalé 13	*An. gambiae* (*s.l.*)	Côte d’Ivoire	Established in LSTM from field collections in conjunction with CSRS (Centre Suisse de Recherches Scientifiques en Côte d’lvoire) [39]	2013
Banfora M	*An. coluzzii*	Burkina Faso Banfora M district	Established in LSTM from field collections in conjunction with CNRFP (Centre National de Recherche et de Formation sur le Paludisme)	2015
VK7 2014	*An. coluzzii*	Burkina Faso Valley de Kou 7	Established in LSTM from field collections in conjunction with CNRFP	2014
FANG	*An. funestus* (*s.s.*)	Colueque, Southern Angola	Supplied by NICD	2015
FUMOZ-R	*An. funestus* (*s.s.*)	Mozambique	Supplied by NICD	2012

^a^MR4 is the Malaria Research and Reference Reagent Resource Centre (https://www.beiresources.org/About/MR4.aspx)

The FANG and FUMOZ-R strains of *Anopheles funestus* were both obtained from the National Institute for Communicable Diseases (NICD), Johannesburg, South Africa. The susceptible FANG colony was colonised in 2002 from Colueque, Southern Angola [15, 16]. The FUMOZ-R strain was colonised from southern Mozambique in 2000, then selected with 0.1% lambda-cyhalothrin to generate a highly resistant strain, FUMOZ-R [16].

### Mosquito rearing

All mosquito strains are maintained in the Liverpool Insect Testing Establishment (LITE) insectaries at LSTM, at 26 ± 2 °C and a relative humidity (RH) of 80 ± 10% under a L12:D12 h light:dark cycle with a 1-h dawn and dusk. Eggs are hatched by adding 2 ml of a 2% brewerʼs yeast slurry to 500 ml of purified (Milipore, Watford, UK) water. Originally, on the day following hatching, first instar larvae were split into small trays of ~ 200 larvae in 500 ml water (0.4 larvae/ml). To facilitate the production of mosquitoes in greater numbers for testing, from March 2017 onwards, larvae were reared in large trays between ~ 600–1000 larvae in 2.5 l depending on the strain (0.24–0.4 larvae/ml).

Larvae were reared in purified water and fed ground fish food (TetraMin tropical flakes, Blacksburg, VA, USA) according to a validated feeding schedule, which varies between species but is consistent between generations. Larval feeding regimes are as follows: for *An. funestus* day 1 (day of hatching) ~ 100 µg/larvae; then day 2–7 ~ 200 µg/larvae; then day 8–10 ~ 233 µg/larvae with day 10 being the first day of pupation; for *An. gambiae* (*s.l.*), depending on the strain, day 1–3 ~ 167–200 μg/larvae; then day 4–8 ~ 200–333 μg/larvae with day 8 being the first day of pupation. Larvae are then fed daily as required until the end of pupation.

Pupae were added to BugDorm-1 rearing cages (Bioquip, Rancho Dominguez, CA, USA) for emergence and adults maintained on 10% sucrose solution fed *ad libitum* from a cotton wick. For egg production, female adults were fed using a Hemotek Membrane Feeding System (Hemotek Ltd., Blackburn, UK). Human blood procured from the non-clinical blood product stock from the blood bank was used until November 2016. Adult mosquitoes were then maintained on horse blood supplied by TCS Biosciences until October 2017. After this point blood supply was switched to blood plasma and red blood cells provided by the human blood bank (mixed upon arrival to LSTM).

One-day post-blood meal an oviposition cup was added to the cages to collect eggs, in purified water in the case of most strains and on wet filter paper in the case of *An*. *funestus* strains. Eggs were treated with 1% bleach on the day they were collected from the cage to remove surface contamination, with eggs hatching the following day [13].

Cohorts of 20 females were weighed before testing and only used if the weight falls within the set thresholds (± 1 standard deviation of the average weight of the colony).

### Colony maintenance: selection and profiling

Insecticide resistant mosquito strains are maintained under selection pressure to preserve their resistant phenotype. Two to five-day-old pyrethroid resistant strains are routinely selected every 3rd generation with 0.05% deltamethrin papers using the WHO susceptibility bioassay [17]. Insecticide papers were purchased from the WHO facility at the Universiti Sains Malaysia (USM), Penang, Malaysia and used a maximum of 6 times. Selection was undertaken at the adult stage as the strains were primarily used to screen for adulticides. If the mortality was less than 10% after exposure then routine selection was extended to every 5th generation; if mortality rose above 10% from 5th generation selections, then testing reverted to every 3rd generation. Exposure times of 1 h for FUMOZ-R and Tiassalé 13, 2 h for VK7 2014 and 3 h for Banfora M were used to ensure at least 20% survival; all adults from the generation to be selected are exposed, with results scored from at least 100 individuals.

All strains are profiled annually against six insecticides, representing the major classes of insecticides currently used for mosquito control, to monitor the stability of their resistance phenotype. Two to five-day-old female mosquitoes are exposed to the WHO diagnostic dose of insecticides and mosquitoes are held in a cabinet maintained at 26 ± 2 °C and 80 ± 10% RH and under a L12:D12 h light: dark cycle until mortality rates were recorded 24 h post-exposure. All papers and test kits are supplied by USM (Table 2). In 2016, the use of bendiocarb papers was discontinued due to inconsistent results and propoxur papers were introduced as a replacement for carbamate resistance profiling. Results from the profiling are interpreted according to the WHO test procedures for insecticide resistance monitoring [17] with Abbottʼs formula [18] used to adjust for control mortality when needed.

**Table 2:** Insecticide, concentration (%) and exposure time used for profiling

Insecticide	Class of insecticide	% concentration	Profiling exposure time (h)
Permethrin	Pyrethroid type I	0.75	1
Deltamethrin	Pyrethroid type II	0.05	1
Fenitrothion	Organophosphate	1	2
Bendiocarb	Carbamate	0.1	1
Propoxur	Carbamate	0.1	1
Dieldrin	Organochlorine	4	1
DDT	Organochlorine	4	1

### Dose response bioassays

The intensity of resistance in the different strains was evaluated using two bioassays: topical application and tarsal (glass plate) exposure. Using these two bioassays allows for a comparison of topical application *vs* tarsal contact to demonstrate the potential of cuticular barriers to insecticide uptake on a tarsal test. Technical grade insecticides were purchased from Greyhound Chromatography and Allied Chemicals (Birkenhead, UK) or Sigma-Aldrich (Poole, UK) and 1% stock solutions were prepared by the addition of HPLC grade acetone (Fisher Chemical, Loughborough, UK). Further serial dilutions were prepared and stored at 4 °C for a maximum of 3 days.

Topical testing: mosquitoes were anesthetized for 30 s with CO_2_ and placed onto a 4 °C chill table (BioQuip Products, Rancho Dominguez, CA, USA). Mosquitoes were carefully turned over using a soft-tipped artists brush to expose the dorsal thorax and to separate them for ease of application. A droplet of 0.25 µl of insecticide in acetone was applied to the dorsal thorax using a 1 cc syringe and a hand-operated micro applicator (Burkhard Scientific, Uxbridge, UK). Following application, mosquitoes were transferred to paper cups and supplied with a 10% sucrose solution and held at 26 ± 2 °C until knockdown was recorded 30 min post-application. RH was not controlled during the application or 30 min post-application recovery phase. Paper cups were then transferred to a stability cabinet and mosquitoes held at 26 ± 2 °C, 70 ± 10% RH until mortality was recorded 24 h post-application. Initial range finding investigations were performed using batches of 10 mosquitoes exposed to a minimum of seven concentrations of insecticides. A set of doses (minimum 5) that resulted in mortalities between 0–100% were selected for further testing using three replicates of 10 individuals for each dose. Three control treatments (with 10 mosquitoes each) of acetone only applications were run for each test that was performed, to give a total of 30 individuals.

Tarsal Testing: glass Petri dishes (radius 2.5 cm, area 19.6 cm^2^) purchased from SLS (Nottingham, UK) were coated with 500 µl of insecticide solution and transferred to an orbital shaker for a minimum of 15 min to allow the solvent to evaporate. Plates were stored at 4 °C for a maximum of 7 days. However, testing was generally performed 4 h after coating with the plates being discarded after use. Plastic deli pots (Cater 4 you Ltd, High Wycombe, UK), into which a hole for transfer of mosquitoes had been introduced, were placed on top of glass plates. Replicates of 10 female mosquitoes were aspirated onto each glass plate and the hole was covered with Parafilm “M” laboratory film. Exposure time was 30 min, following which the mosquitoes were aspirated to paper cups and supplied with 10% sucrose solution and the initial knockdown effect was scored. Bioassays were conducted at 26 ± 2 °C and the humidity was not controlled during the test. Paper cups were then transferred to a cabinet maintained under the environmental conditions described above and mortality was recorded 24 h post-application. Initial range-finding experiments were performed before selecting a minimum of five concentrations. Three replicates of 10 mosquitoes were tested at each concentration and acetone-only controls were performed as described above. In addition, rapeseed oil methyl esters (RME) (Bayer AG, Monheim, Germany) was included to determine the efficacy of permethrin uptake with the addition of this adjuvant [12]. RME was diluted to 0.39 mg/ml in HPLC grade acetone and this RME acetone solution was used to prepare insecticide dilutions. Tarsal assays with RME were performed as described above.

Bioassay observations of 24-h mortality were subjected to the Pearsonʼs goodness-of-fit-chi-square test and probit analysis with PoloPlus 2.0 (LeOra software, El Cerrito, CA, USA) to estimate LC_50_ or LD_50_ values. Topical testing data was converted from lethal concentrations (LC) into lethal doses (LD), expressed as µg of insecticide per mg of mosquito. If control mortality was < 20% but ≥ 5% then the observed mortality was corrected using Abbott’s formula [18].Where control mortality was > 20% the results were discarded and the test replicate repeated.

### Genotyping

For quality control purposes, and to monitor for the stability of resistance mechanisms in the strains, each colony is genotyped approximately every 5th generation to determine species and the frequency of known target site resistance alleles.

Three different *kdr* alleles were screened for initially (1014S, 1014F and 1575Y) plus the *ace-1* G119S allele; strains were then routinely screened for the mutations identified as being present.

For each round of genotyping, genomic DNA was extracted from 48 non-blood-fed females using a Qiagen blood and tissue DNA extraction kit. Species ID was performed on *An. gambiae* (*s.l.*) strains using the method described by Scott et al. [14], followed by a restriction enzyme digest to distinguish between *An. gambiae* (*s.s*) and *An. coluzzii* [19]. For *An. funestus* species ID, the method described by Cohuet et al. [20] was used. *Kdr* alleles were detected using Taqman™ assays [21, 22]. Genotyping of the G119S (*ace-1*) mutation in *An. gambiae* was carried out using the TaqMan™ method described by Bass et al. [23].

### Synergist bioassays

The WHO synergist bioassay is used to assess the contribution of metabolic resistance mechanisms to the observed resistance phenotypes. In this case, PBO was used to screen for the involvement of cytochrome P450 monooxygenases (P450s) in conferring pyrethroid resistance. Tests were performed according to the WHO protocol [17] with a 1-h pre-exposure of 2–5 day-old adult mosquitoes to papers impregnated with PBO (4%) followed by a 1-h exposure to papers impregnated with permethrin (0.75%). Four controls were used: a negative control blank paper (no treatment), a PBO control, where a 1-h PBO exposure was followed by 1-h blank exposure; a 1-h blank exposure followed by 1-h permethrin exposure and a positive control 2-h exposure to fenitrothion (1%). The Fisherʼs exact probability test, one-tailed, was used to determine the significance of the synergistic effect of PBO. The total number, across 3 replicates, of mosquitoes alive and dead 24 h after exposure to permethrin with or without a pre-exposure to PBO was included in the pairwise comparison.

### Quantification of resistance-associated gene expression

RNA was extracted from three pools of 5–7, 2–5-day-old females using a PicoPure RNA isolation kit (Thermo Fisher Scientific, Warrington, UK). One to four μg of RNA from each biological replicate was reverse transcribed using Oligo dT (Invitrogen, Warrington, UK) and Superscript III (Invitrogen). The resulting cDNA was diluted to 4 ng/µl and used as a template in the subsequent PCR reactions. Primers and probes as described by Maviridis et al. [24] were ordered from Integrated DNA Technologies (Leuven, Belgium), with Cy5 replacing Atto647N. Primers and probes were diluted to 10 µM for use in a 10 µl final reaction. Four multiplex reactions were carried out on each cDNA set in technical triplicate, as follows: (i) *CYP6P4*, *CYP6Z1* and *RPS7*; (ii) *CYP4G16* and *CYP9K1*; (iii) *CYP6M2* and *CYP6P1*; (iv) *CYP6P3* and *GSTE2*. PrimeTime Gene Expression Master Mix (Integrated DNA Technologies) was used to set up each reaction following the manufacturerʼs instructions. Each reaction was carried out on a MxPro 3005P qPCR System (Agilent) with the following thermocycling conditions: 3 min at 95 °C followed by 40 cycles of 15 s at 95 °C; 1 min at 60 °C. Cycle threshold (Cq) values were exported and analysed using the ΔΔct methodology [25], using RPS7 as an endogenous control. Each resistant population was compared to the susceptible Kisumu population and the fold change reported.

The expression levels of additional, newly identified, candidate insecticide resistance genes; an alpha crystalline (AGAP007161) and an ATPase subunit (AGAP006879) [9] and *SAP2* (AGAP008052) [10] were also determined by SYBR Green qPCR using cDNA (2 ng/µl), extracted as described above, and using previously published PCR primers [19]. Each 20 µl reaction contained 10 µl of SYBR Green Supermix (Agilent, Stockport, UK), 0.3 µM forward and reverse primer and 1 µl of cDNA. qPCR was performed under the following conditions: 3 minutes at 95 °C, with 40 cycles of 10 s at 95 °C and 10 s at 60 °C; EF and S7 were used as endogenous controls as in [19]. Delta ct (Δct) values were used to test for significant upregulation or downregulation of metabolic genes compared to Kisumu. A homogeneity of variance test was used to determine if data were normally distributed. Δct values were transformed to normalise (where applicable) and an ANOVA test, followed by Dunnettʼs test was performed. Where transformations did not normalise the data, a Dunn test was performed.

## Results

### Selection

Pyrethroid resistance in Tiassalé 13, FUMOZ-R and VK7 2014 remained stable across all generations tested. (Additional file 1: Figure S1). The Banfora M colony was not maintained in LITE beyond July 2017 and hence selection data are not reported.

### Profiling

**Fig. 1:**
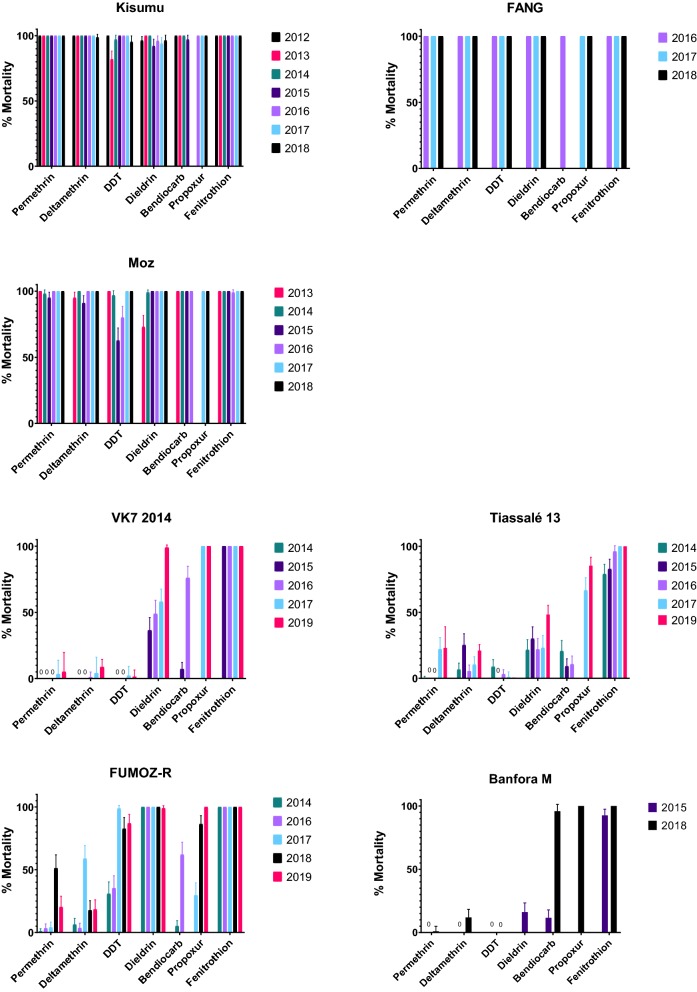
WHO susceptibility profiling. Mortality rates (%) 24 hours after exposure for 7 strains of *Anopheles* mosquito. Error bars represent 95% binomial confidence intervals

Results of profiling the seven strains of anopheline mosquitoes with discriminating dose assays are shown in Fig. 1. The 95% binomial confidence intervals for the whole population are displayed. Within each set of replicates in each testing round, the standard deviations were < 20% in all cases with the exception of FUMOZ-R 2017 deltamethrin (21.39%) and 2018 and 2019 permethrin (20.87% and 26.19%, respectively).

FANG, Kisumu, and Moz were fully susceptible to all insecticides; this susceptibility was stable across all generations, although in some rounds of testing, a low prevalence of resistance to DDT (81–100% and 62–100% mortality) and dieldrin (92–100% and 73–100% mortality) was detected in Kisumu and Moz, respectively. Tiassalé 13 and VK7 2014 are resistant to pyrethroids and DDT and this resistance remained stable over all generations. Both strains were also initially resistant to dieldrin although this resistance has now been lost in VK7 2014. Low levels of fenitrothion resistance were present in Tiassalé 13 initially but this resistance was lost over time. Results from carbamate bioassays are harder to interpret; initial results with bendiocarb indicated a high prevalence of resistance in Tiassalé 13 and VK7 2014; however, higher mortalities were later seen when the carbamate used for profiling was switched from bendiocarb to propoxur. In 2015 Banfora M had confirmed resistance to all insecticides. In 2018 pyrethroid and DDT resistance was still present, but, carbamate resistance had dramatically reduced (12% mortality in 2015 and 96% in 2018). The low level of organophosphate resistance seen in 2015 has also been lost over time.

Resistance was less stable in FUMOZ-R; this population was resistant to pyrethroids at all time points tested but resistance to carbamates and organochlorines has declined over time with the latest results suggesting FUMOZ-R is fully susceptible to propoxur and dieldrin (100% and 99% mortality, respectively).

### Dose-response bioassays

**Fig. 2:**
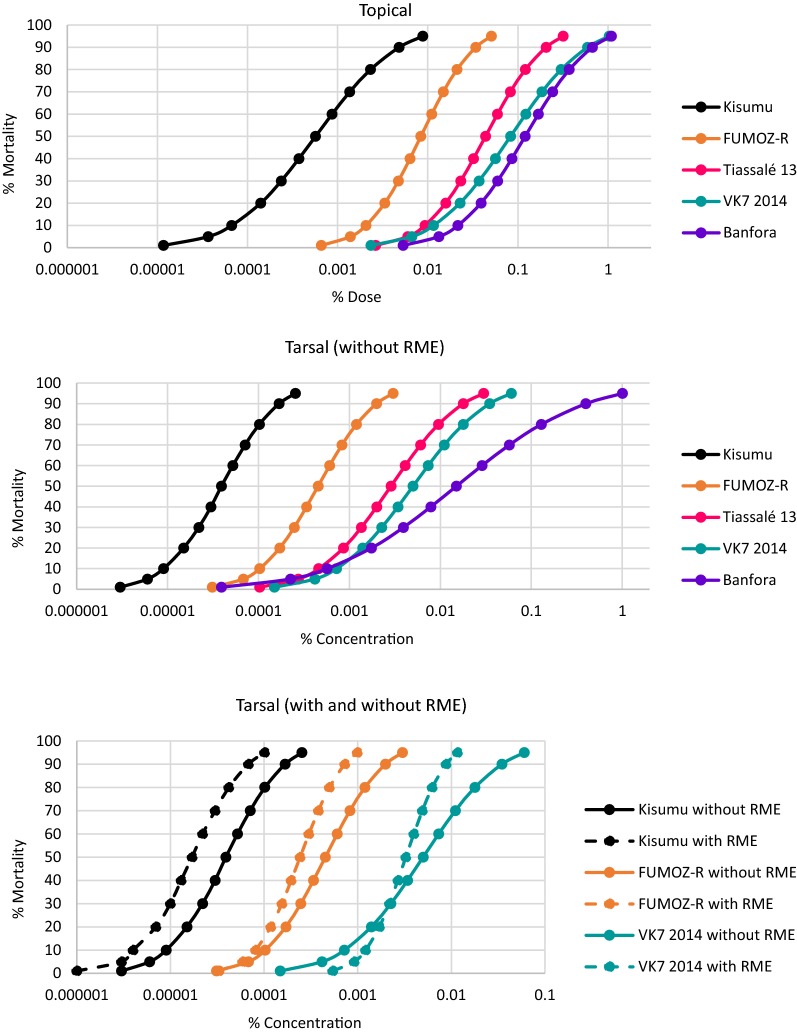
Permethrin dose-response curves for topical (lethal dose) and tarsal bioassays with and without RME (lethal concentration). Mortality rates are recorded 24 hours post-exposure

The dose response curves for permethrin with topical, tarsal testing without RME and tarsal testing with RME are shown in Fig. 2. Resistance ratios (RRs) were calculated by dividing the LC_50_ of the resistant population by the LC_50_ of the susceptible Kisumu strain and are shown in Table 3. All four resistant strains were significantly more resistant to pyrethroids than Kisumu in both bioassays. The Banfora M strain was the most pyrethroid resistant followed by VK7 2014, Tiassalé 13 and FUMOZ-R in both topical application and tarsal contact assays. RRs were similar between the two bioassay techniques for all strains except for Banfora M where the tarsal exposure RR was 1.7 times higher than in the topical application bioassay.

**Table 3:** Topical and tarsal resistance ratios (RRs) relative to Kisumu and 95% confidence interval (CI)

RRs	FUMOZ-R	Tiassalé 13	VK7 2014	Banfora M
Topical	14.74	76.91	145.77	222.48
95% CI	16.4–36.9	84.6–195	149–397	205–511
Tarsal	11.49	73.33	128.23	384.51
95% CI	7.8–17.0	43.0–122.8	81.4–198.5	21.4–6781
Tarsal/Topical ratio	0.78	0.95	0.88	1.73

The addition of the adjuvant RME improved the efficacy of permethrin against Kisumu, FUMOZ-R and VK7 2014 with an LC_50_ fold change decrease of 2.29, 1.86 and 1.53, respectively. The RME with permethrin tarsal test was not performed for Tiassalé 13 or Banfora M. The addition of RME reduced the resistance ratios (to Kisumu without RME) for FUMOZ-R and VK7 2014 (RR of 6.2 for FUMOZ-R + RME *vs* Kisumu, 11.6 for Fumoz-R *vs* Kisumu; RR of 83.8 for VK7 2014 + RME *vs* Kisumu, 128.2 for VK7 2014 *vs* Kisumu).

### Target site/point mutation genotyping

Species ID PCRs confirmed Kisumu as *An. gambiae* (*s.s.*) (formerly S form), VK7 2014 and Banfora M as *An. coluzzii* (formerly M form), Moz as *An. arabiensis*, and FANG and FUMOZ-R as *An. funestus* (*s.s*.) Over time species ID has been confirmed 12 times for Kisumu and Moz, 10 times for FUMOZ-R, 8 times for VK7 2014, and 4 times for FANG and Banfora M, and there has been no evidence of contamination. For the Tiassalé 13 strain, only *An. coluzzii* was detected when the strain was first colonised from the field but over 11 subsequent rounds of genotyping, *An. gambiae* (*s.s*.) was also detected and the proportion of *An. gambiae* (*s.s*.) individuals increased to reach 98% (with the remaining 2% being hybrid) in the last round of genotyping in November 2018 (Additional file 2: Figure S2). The same shift from *An. coluzzii* to *An. gambiae* was seen for a previously established Tiassalé colony (Tiassalé 2) colonised in July 2011 and maintained until June 2014 (Additional file 2: Figure S2).

**Fig. 3:**
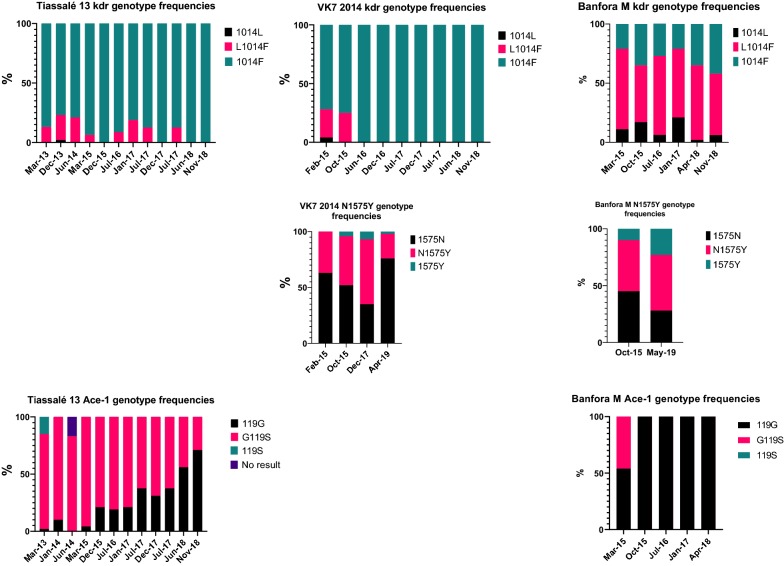
Frequency of *kdr*, N1575Y and *ace-1* genotypes in *An. gambiae*. 1014L, 119G and 1575N indicate the wildtype allele. 1014F, 119S and 1575Y indicate the resistant allele

The three pyrethroid resistant strains of *An. gambiae* (*s.l*.) were screened for *kdr* mutations (Fig. 3). The 1014S mutation was not detected in any strain. The frequency of the 1014F allele was high (> 80%) in all tests for Tiassalé 13 and VK7 2014 and, in the most recent round of genotyping, the 1014F allele was fixed in both populations (100%). L1014F heterozygotes predominated in the Banfora M strain, and in the latest round of genotyping the 1014F allele frequency was 60%. The 1575Y *kdr* allele was detected in both VK7 2014 and Banfora M but not in Tiassalé 13.

Two of the *An. gambiae* (*s.l.*) strains contained a low frequency of the *ace-1* 119S allele on colonisation but the frequency of this resistance allele decreased rapidly in the absence of selection with insecticides targeting this enzyme (organophosphates or carbamates) and is now absent in Banfora M (Fig. 3). *Kdr*, *ace-1* and N1575Y allele frequencies from the most recent round of genotyping for Tiassalé 13, VK7 2014 and Banfora M are available in Additional file 3: Table S1. To date, no knockdown resistance (*kdr*) mutation in the voltage-gated sodium channel gene has been reported in *An. funestus* (*s.s.*) Africa-wide and so FUMOZ-R was not included in genotyping.

### PBO synergism bioassays

**Fig. 4:**
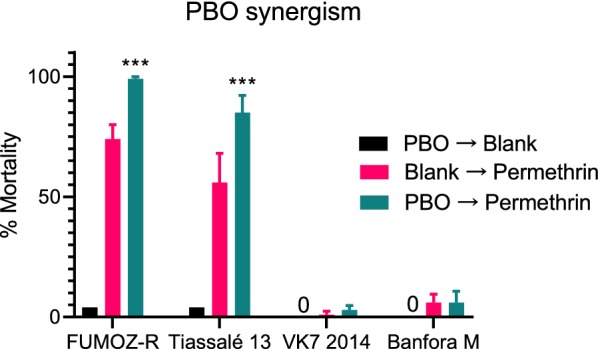
PBO synergism results for four resistant anopheline strains. Mortality is expressed as a % from four tubes of ~25 mosquitoes; error bars represent standard error; statistical differences between permethrin only and PBO + permethrin are indicated as ***P < 0.001

The very low level of mortality 24 h after a one-hour exposure to permethrin was not significantly increased by pre-exposure to PBO in Banfora M or VK7 2014 (*P* > 0.05) (Fig. 4), indicating that other potent resistance mechanisms are present in this strain. A significant (*P* < 0.001) synergistic effect of PBO was however seen in Tiassalé 13 (mortality increased from 56% to 85%) and FUMOZ-R (mortality increased from 74% to 99%) indicating a key role of cytochrome P450-mediated resistance in these strains. Negative controls (both control papers only or control papers followed by PBO) and positive (Fenitrothion) controls gave < 4% and 100% mortality in each case, respectively.

### Metabolic resistance: P450 expression levels

**Fig. 5:**
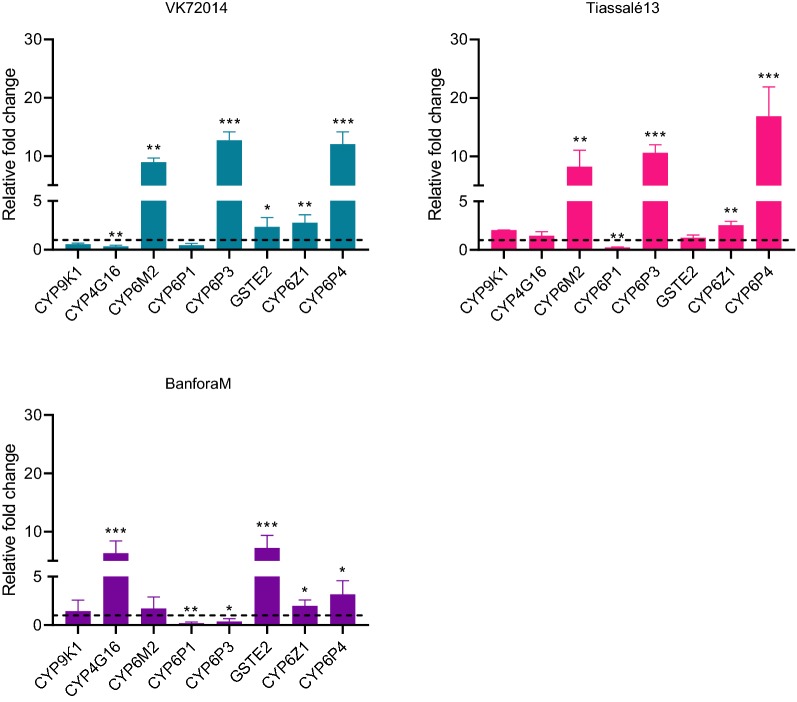
P450 expression in three resistant strains normalised to Kisumu. Error bars represent standard deviations, statistically significant differences in expression level relative to Kisumu are indicated as *P < 0.05, **P < 0.01, ***P ≤ 0.001

Expression levels of P450s in resistant strains were compared to the susceptible strain Kisumu and the relative expression level was reported as a fold change (Fig. 5). Upregulation (more than 8-fold) of *CYP6M2, CYP6P3*, and *CYP6P4* was seen in Tiassalé 13 and VK7 2014, these P450s are known to metabolise pyrethroids [26, 27]. Banfora M had upregulation (more than 6-fold) of the glutathione S-transferase GSTE2 (a DDT metaboliser [28]) and the P450 *CYP4G16*, involved in cuticular hydrocarbon synthesis [8].

### *SAP2* alpha crystallin and ATPase

**Fig. 6:**
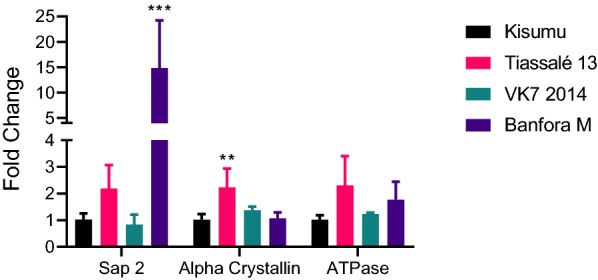
*SAP2* (AGAP008052), alpha crystallin (AGAP007161) and ATPase (AGAP006879) expression in three resistant strains and Kisumu. Error bars represent standard deviations. Statistically significant differences in expression level relative to Kisumu are indicated as **P < 0.01, ***P ≤ 0.001

The expression of three additional resistance candidates [9, 10] was measured in three of the pyrethroid resistant strains (Tiassalé 13, VK7 2014 and Banfora M) and compared with the susceptible Kisumu strain (Fig. 6). The alpha crystallin (AGAP008052) was significantly upregulated in Tiassalé 13 and *SAP2* was highly upregulated in Banfora M.

## Discussion

Screening of new insecticide candidates against a range of stable characterised populations of the target species forms a pivotal role in the product development pathway, enabling potential cross-resistance risks to be identified at an early stage. LITE strives to maintain a range of strains that include the major resistance mechanisms that are thought to be of major operational significance in Africa. The present paper describes the resistance profiles of the strains that LITE currently maintains and their underpinning mechanisms.

Three susceptible mosquito strains are maintained from three different species of African malaria vectors, *An*. *gambiae* (*s.s.*) (Kisumu), *An. arabiensis* (Moz) and *An. funestus* (FANG). Resistant *Anopheles* strains currently encompass three species: *An. gambiae* (*s.s.*), *An. coluzzii* and *An. funestus.* Two strains of *Ae. aegypti* populations (New Orleans and Cayman) are also maintained in LITE and although not described in detail in this manuscript, information on insecticide profiling and genotyping is provided in Additional file 4: Figure S3 (*kdr* allele frequencies) and Additional file 5: Figure S4 (*Aedes* colony profiling).

Selection with the pyrethroid deltamethrin was chosen to maintain high levels of pyrethroid resistance in the resistant strains, given the primacy of this insecticide class in malaria vector control. Selecting with deltamethrin every 3rd to 5th generation has ensured that pyrethroid resistance has remained relatively stable over time. However, resistance to other insecticide classes, that may have been present at the time of colonisation, notably carbamates and organophosphates, have been lost from these strains. Is it important to note however, that interpretation of the carbamate bioassay data is complicated by the change from bendiocarb to propoxur and the issue of differential susceptibility to insecticides within classes warrants further study. With the recent move away from pyrethroids for IRS, we have attempted to identify and colonise strains with resistance to other chemistries formulated into IRS products. However, to date, attempts to establish and maintain pirimiphos-methyl resistant populations in our insectaries have been unsuccessful.

Banfora M and Tiassalé 13 are both resistant to the cyclodiene dieldrin, despite the fact that this insecticide has not been used for malaria control since the 1960s. This resistance was associated with an alanine to glycine substitution in the Rdl locus of *An. gambiae* (*Rdl* allele) [29] and remained relatively stable in Tiassalé 13 (data not shown) despite the absence of selection pressure.

### Resistance mechanisms

The 1014F *kdr* allele is present at high levels in two of the *An. gambiae* (*s.l.*) strains (Tiassalé 13 and VK7 2014) but was found at much lower levels in the Banfora M strain. In the latter strain the frequency of the 1014F allele is comparatively low (68%) *vs* VK7 2014 & Tiassalé 13 and surprisingly did not increase following pyrethroid selection. The 1575Y *kdr* allele was found in both of the strains from Burkina Faso (VK7 2014 and Banfora M) but again, did not appear to increase in frequency in response to selection with deltamethrin. Recently, several supplementary amino acid substitutions have been identified in the sodium channel of resistant *An. gambiae* populations [30] but the majority of these have not yet been shown definitively to be associated with resistance and we have not yet genotyped the LITE strains for these mutations. The *ace-1* 119S allele was present in two of the strains (Tiassalé 13 and Banfora M) on colonisation but the frequency of this decreased rapidly in the absence of selection with insecticides targeting the acetylcholinesterase enzyme; this mutation is no longer detectable in the Banfora M strain and is now only present in the heterozygous form in Tiassalé 13. The reduction in frequency of *ace-1* mirrors the loss of resistance to organophosphates and carbamates in these populations after prolonged colonisation.

Metabolic resistance to pyrethroids is implicated by PBO synergism assays in FUMOZ-R and Tiassalé 13 and by quantitative PCR showing the overexpression of P450 genes encoding enzymes with known pyrethroid metabolism activity (*CYP6M2*, *CYP6P3* and *CYP6P4* [31]) in VK7 2014 and Tiassalé 13. Again, the Banfora M population appears to be more distinct from the other pyrethroid resistant strains with the largest differences in gene expression observed for the glutathione S-transferase *GSTE2* and the P450 *CYP4G16*. GSTE2 metabolises DDT [28] and the ortholog in *An. funestus* has been shown to metabolise pyrethroids [32]. CYP4G16 catalyses the final step in the pathway of cuticular hydrocarbon synthesis [33] and knockdown of *CYP4G16* in *Anopheles* results in lower amounts of cuticular hydrocarbons and decreased tolerance to desiccation [34]. The elevated expression of *CYP4G16* in Banfora M may be indicative of a cuticular or penetration barrier resistance mechanism, as discussed below. Although we did not characterise the molecular mechanisms underpinning pyrethroid resistance in FUMOZ-R in this study, previous molecular analyses have revealed high overexpression of the duplicated cytochrome P450 genes *CYP6P9a* and *CYP6P9b* in this strain, and the high level of PBO synergism we observed is supportive of P450s being the dominant resistance mechanism in this strain [35, 36].Other less well characterised resistance mechanisms, for which molecular diagnostics are not available, may be contributing to the pyrethroid resistance phenotype in our strains. Indeed, when we tested a small subset of genes recently implicated in pyrethroid resistance from a meta-analysis of transcriptomic data on resistance strains from across Africa we found very high levels of expression of *SAP2*, a pyrethroid binding protein found in the legs of resistant mosquitoes in the Banfora M strain [10].

### Comparing pyrethroid resistance levels between strains

We performed quantitative bioassays on our resistant lines in order to establish the strength or intensity of resistance in our four pyrethroid resistant strains. Additional insecticide topical and tarsal bioassays were run to determine resistance intensity of bendiocarb, DDT and pirimiphos-methyl (Additional file 6: Table S2). Using both tarsal and topical assays, Banfora M was the most resistant to permethrin followed by VK7 2014, Tiassalé 13 and FUMOZ-R although the resistance ratio for VK7 2014 had overlapping confidence intervals with both Tiassalé 13 and Banfora M. Inclusion of the adjuvant RME prevents insecticides from crystallising on a glass surface and seems to improve uptake of some insecticides through the cuticle of exposed insects [12]. Here RME improved the efficacy of permethrin against Kisumu, FUMOZ-R and VK7 2014. For three of the strains, the resistance ratio when compared to the susceptible Kisumu strain was lower for tarsal testing than topical. However, for Banfora M, a 1.73-fold higher resistance ratio was detected for tarsal compared to topical. A reduction in resistance when insecticides are applied topically directly in solvent, suggests that barriers to penetration are contributing to the resistance phenotype [37] in the Banfora M strain. This is supported by the molecular data described above. Further work is ongoing to establish the role and mechanisms of penetration resistance in this strain.

### A strange case of species displacement

Species ID was used as one means of checking for contamination between strains. Additional genotyping was performed between 2011–2014 to look for extra diagnostic SNPs; however, none were found to be discriminating (Additional file 7: Table S3). As anticipated, given LITE’s measures to avoid cross-contamination between mosquito strains, species remained constant across all generations for all strains with the exception of the Tiassalé 13 colony. Females used to establish this colony were confirmed as *An. coluzzii*, as were a random subset of six progeny from each, prior to pooling into a single colony. However, nine months later the majority of the individuals tested were hybrids between *An. gambiae* and *An. coluzzii*, and, in subsequent genotyping rounds, a high proportion of the samples were *An. gambiae* (*s.s*.) By 2018 the colony was 98% *An. gambiae* (*s.s.*) This apparent change in species composition had been observed in a previous strain colonised from Tiassalé, Tiassalé 2 established in July 2011. Again, at the point of colonisation 100% of samples tested were *An. coluzzii*. This colony was eventually discarded in June 2014, and replaced with the current Tiassalé 13, as we feared that a contamination event had likely occurred when 100% of samples tested were identified as *An. gambiae* (*s.s.*) The reason for this shift from *An. coluzzii* to *An. gambiae* is unknown but is unlikely to be explained by a rearing contamination event in either case. The only other *An. gambiae* strain held in LITE, Kisumu, is kept separately from the resistant strains and had either of the Tiassalé strains been contaminated with Kisumu a drop in pyrethroid resistance would have been detected which was not observed in either case. It is possible that a small number of the founder females had been inseminated by *An. gambiae* (*s.s.*) which was not detected in the initial screening of larval progeny and that *An. gambiae* proved better adapted to colony conditions than *An. coluzzii.* However, this goes against our experience of colonisation, and that of others, that typically West African *An. gambiae* (*s.s.*) strains are much harder to maintain in colony than *An. coluzzii.*

## Conclusions

The differences in resistance profiles of the four resistant strains described here highlight the importance of screening new insecticides against a range of resistant populations possessing different mechanisms of resistance. The two Burkina Faso strains, VK7 2014 and Banfora M, have the highest levels of resistance to pyrethroids, but differ in the underpinning mechanisms with resistance in VK7 2014 being largely mediated by *kdr* and P450 overexpression whereas penetration barriers appear to be more important in Banfora M. Tiassalé 13 has lower levels of pyrethroid resistance but has also maintained resistance to DDT and carbamates conferred by a combination of target site mutations and P450s. Resistance in FUMOZ-R is mediated by P450s. Despite the diversity in resistance profiles incorporated in these strains, new resistance mechanisms are continually being selected for in the field through the intensive use of insecticides, both for vector control and for the control of crop pests and hence screening on laboratory strains alone can never be a substitute for evaluating new insecticides against field populations. However, we have shown through our work how screening novel and repurposed chemistries against well-characterised resistant strains and under carefully controlled and standardised conditions can provide an important early stage assessment of cross-resistance risk and it is hoped that we have provided a useful resource for developers in designing compound screening pathways.

## Supplementary information

**Additional file 1: Figure S1.** Selection (0.05% deltamethrin) data over time. WHO tube bioassay 24 hour % mortality.

**Additional file 2: Figure S2.** Proportion of *An. coluzzii* and *An. gambiae* in Tiassalé colonies over time.

**Additional file 3: Table S1.** *kdr*, *ace-1* and N1575Y genotype (%) and allele frequencies from the most recent round of genotyping for Tiassalé 13, VK7 2014 and Banfora M.

**Additional file 4: Figure S3.** Frequency of two amino acid substitutions in the voltage gated sodium channel in the Cayman population, a pyrethroid resistant strains of *Ae. aegypti*, maintained in LITE. The susceptible strain New Orleans (not shown) has remained fully homozygous wildtype since March 2011.

**Additional file 5: Figure S4.** *Aedes aegypti* colony profiling. Mortality rates 24 hours after exposure for 2 strains of *Ae. aegypti*.

**Additional file 6: Table S2.** Topical and tarsal resistance ratios of additional insecticides. *Abbreviation*: ND, not done.

**Additional file 7: Table S3.** Additional genotype (%) and allele frequencies for extra-diagnostic SNPs.

## Data Availability

The datasets generated and/or analysed during the present study are all summarised in the article and full datasets are available from the corresponding author upon reasonable request. Requests for sharing biological material will be subject to ethical and commercial considerations but every effort will be made to accommodate reasonable requests. Standard Operating Procedures for any of the procedures used in this manuscript can be shared with interested parties upon request.

## Abbreviations

LSTM: Liverpool School of Tropical Medicine
IVCC: Innovative Vector Control Consortium
LITE: Liverpool Insect Testing Establishment
DDT: dichlorodiphenyltrichloroethane
PCR: polymerase chain reaction
qPCR: quantitative polymerase chain reaction
Cq: cycle threshold
RME: rapeseed oil methyl esters
kdr: knockdown resistance
ace-1: acetylcholinesterase point mutation (G119S)
PBO: piperonyl butoxide
ΔΔct: delta delta cycle threshold
ANOVA: analysis of variance
