# Supplementary material for: Characterisation of Anopheles strains used for laboratory screening of new vector control products
Source: Parasit Vectors. 2019 Nov 5;12:522. doi: 10.1186/s13071-019-3774-3 (PMC6833243; doi:10.1186/s13071-019-3774-3)
Supplement: Supplementary file 1 — Additional file 1: Figure S1. Selection (0.05% deltamethrin) data over time. WHO tube bioassay 24 hour % mortality. [file 13071_2019_3774_MOESM1_ESM.pdf]

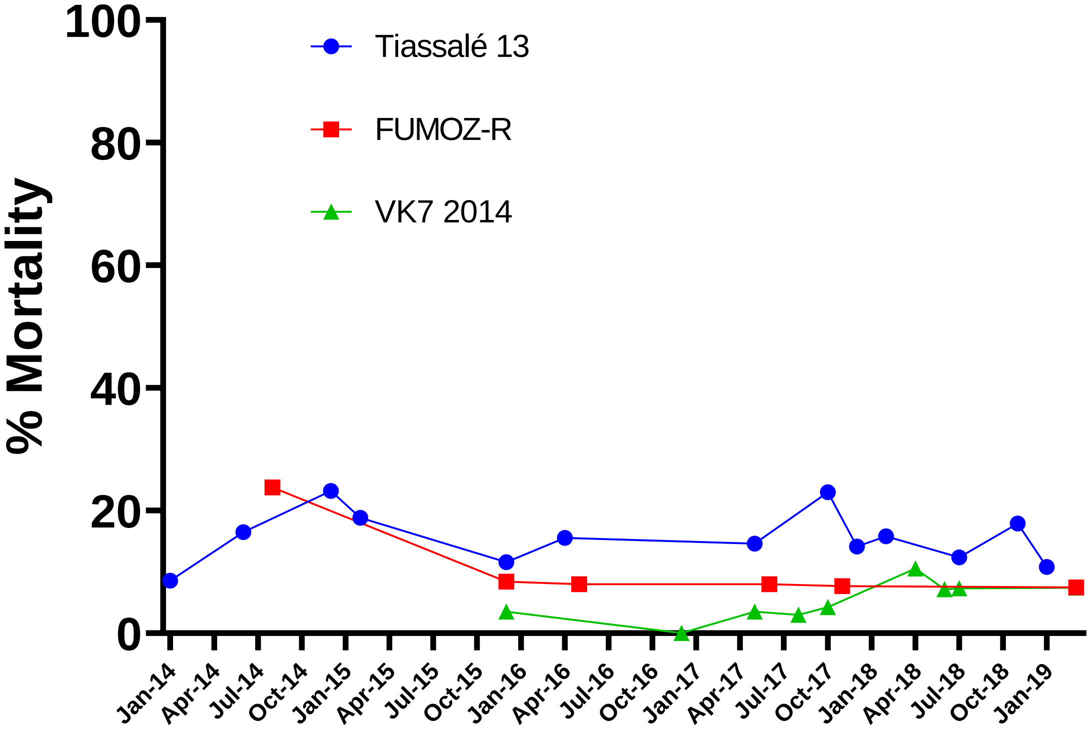

Additional file 1: Figure S1. Selection (0.05% Deltamethrin) data over time. WHO tube bioassay 24 hour % mortality.
