# Supplementary material for: Characterisation of Anopheles strains used for laboratory screening of new vector control products
Source: Parasit Vectors. 2019 Nov 5;12:522. doi: 10.1186/s13071-019-3774-3 (PMC6833243; doi:10.1186/s13071-019-3774-3)
Supplement: Supplementary file 2 — Additional file 2: Figure S2. Proportion of An. coluzzii and An. gambiae in Tiassalé colonies over time. [file 13071_2019_3774_MOESM2_ESM.pdf]

Tiassalé 13 *An. coluzzii* and *An. gambiae* % species proportion

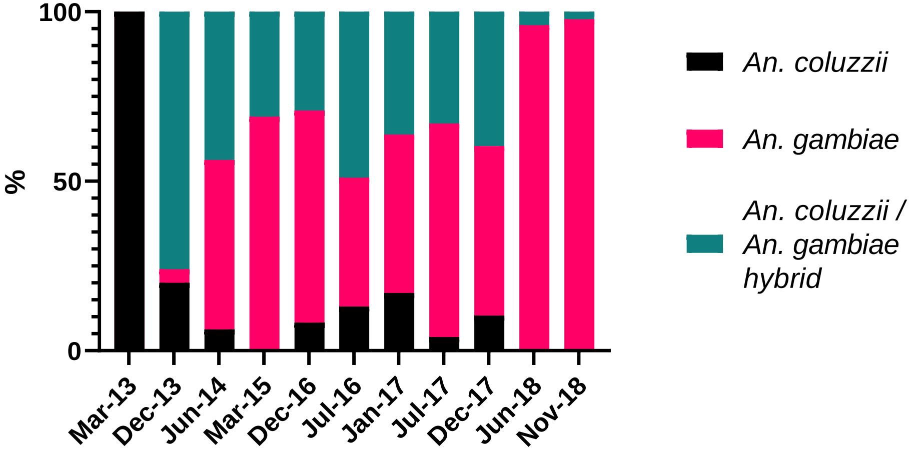

Tiassalé 2 *An. coluzzii* and *An. gambiae* % species proportion

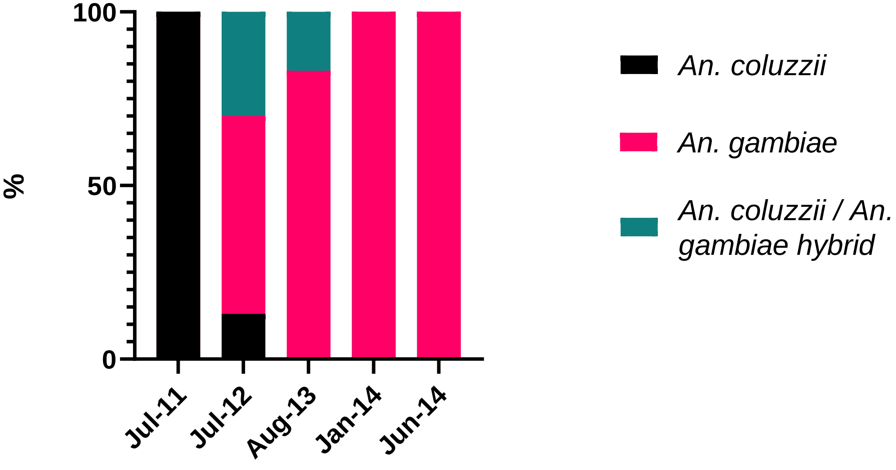

Additional file 2: Figure S2. Proportion of *An. coluzzii* and *An. gambiae* in Tiassalé colonies over time.
