# Supplementary material for: Characterisation of Anopheles strains used for laboratory screening of new vector control products
Source: Parasit Vectors. 2019 Nov 5;12:522. doi: 10.1186/s13071-019-3774-3 (PMC6833243; doi:10.1186/s13071-019-3774-3)
Supplement: Supplementary file 3 — Additional file 3: Table S1. kdr, ace-1 and N1575Y genotype (%) and allele frequencies from the most recent round of genotyping for Tiassalé 13, VK7 2014 and Banfora M. [file 13071_2019_3774_MOESM3_ESM.pdf]

Additional file 3: Table S1. kdr, ace-1 and N1575Y genotype (%) and allele frequencies from the most recent round of genotyping for Tiassalé 13, VK7 2014 and Banfora M.

Strain	Genotype (%)				Date of last screening
	Susceptible	Heterozygote	Resistant		
**Kdr 1014**	**1014L**	**L1014F**	**1014F**	**Allele frequency**	**kdr**
Tiassalé 13	0	0	100	1	Nov-18
VK7 2014	0	0	100	1	Nov-18
Banfora M	21	58	21	0.68	Nov-18
**Kdr 1575**	**1575N**	**N1575Y**	**1575Y**	**Allele frequency**	**N1575Y**
Tiassalé 13	100	0	0	0	Mar-13
VK7 2014	35	58	7	0.35	Dec-17
Banfora M	28	49	23	0.48	May-19
ace-1	**119G**	**G119S**	**119S**	**Allele frequency**	**ace-1**
Tiassalé 13	71	29	0	0.15	Nov-18
VK7 2014	100	0	0	0	Nov-18
Banfora M	100	0	0	0	Apr-18
