# Supplementary material for: Characterisation of Anopheles strains used for laboratory screening of new vector control products
Source: Parasit Vectors. 2019 Nov 5;12:522. doi: 10.1186/s13071-019-3774-3 (PMC6833243; doi:10.1186/s13071-019-3774-3)
Supplement: Supplementary file 4 — Additional file 4: Figure S3. Frequency of two amino acid substitutions in the voltage gated sodium channel in the Cayman population, a pyrethroid resistant strains of Ae. aegypti, maintained in LITE. The susceptible strain New Orleans (not shown) has remained fully homozygous wildtype since March 2011. [file 13071_2019_3774_MOESM4_ESM.pdf]

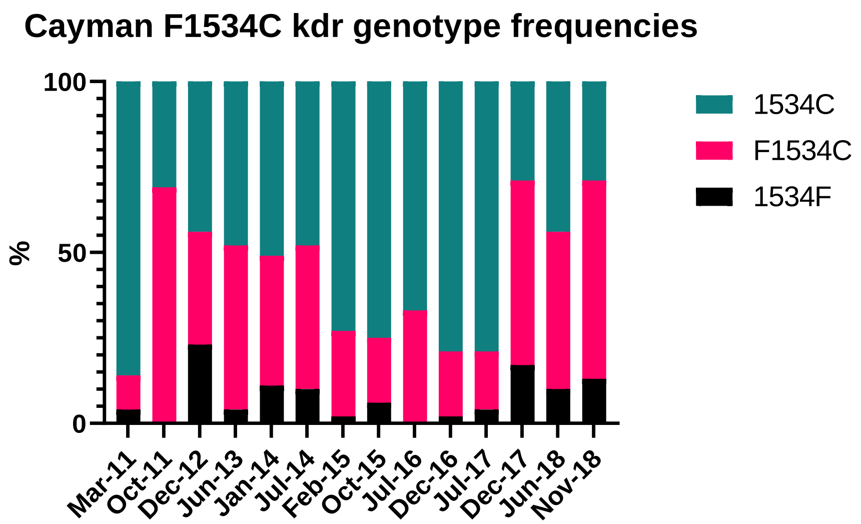


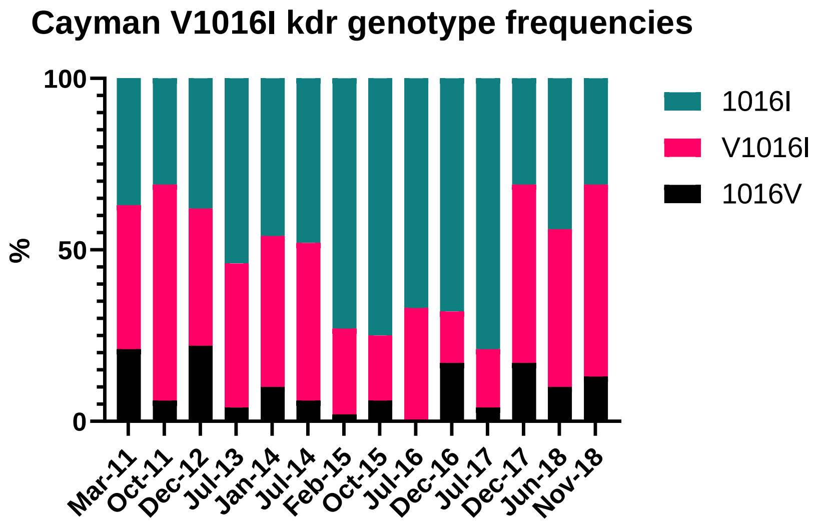


**Additional file 4: Figure S3.** Genotype frequency of two amino acid substitutions in the voltage gated sodium channel in the Cayman population, a pyrethroid resistant strains of *Aedes aegypti*, maintained in LITE. The susceptible strain New Orleans (not shown) has remained fully homozygous wildtype since March 2011.
