# Supplementary figures and images for: Characterisation of Anopheles strains used for laboratory screening of new vector control products
Source: Parasit Vectors. 2019 Nov 5;12:522. doi: 10.1186/s13071-019-3774-3 (PMC6833243; doi:10.1186/s13071-019-3774-3)

## New Orleans

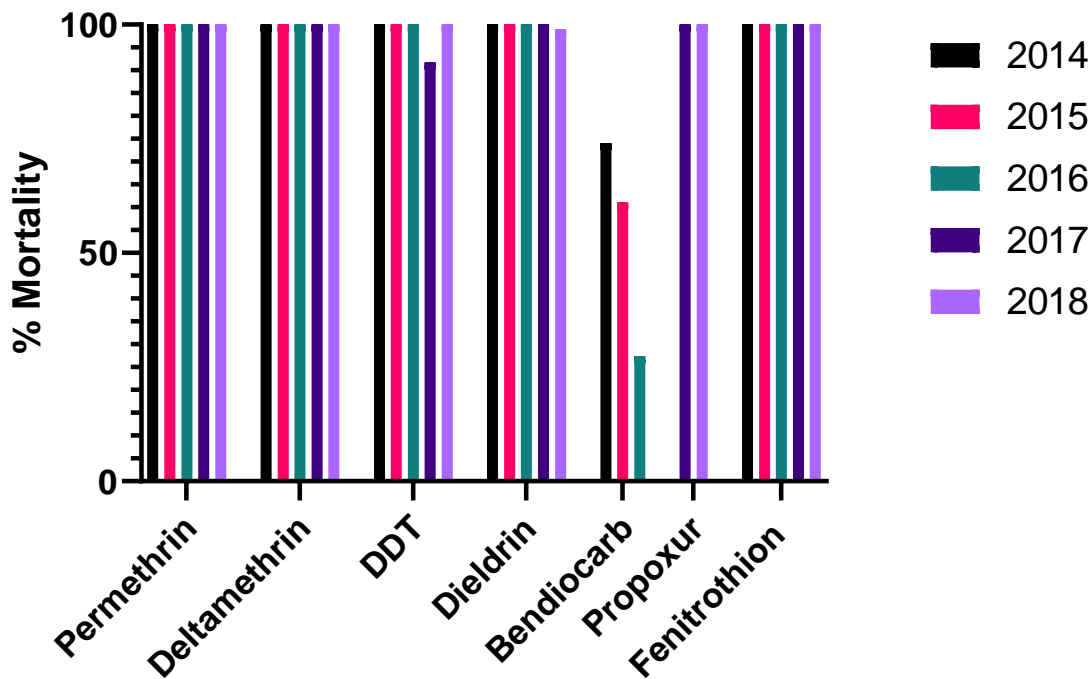

## Cayman

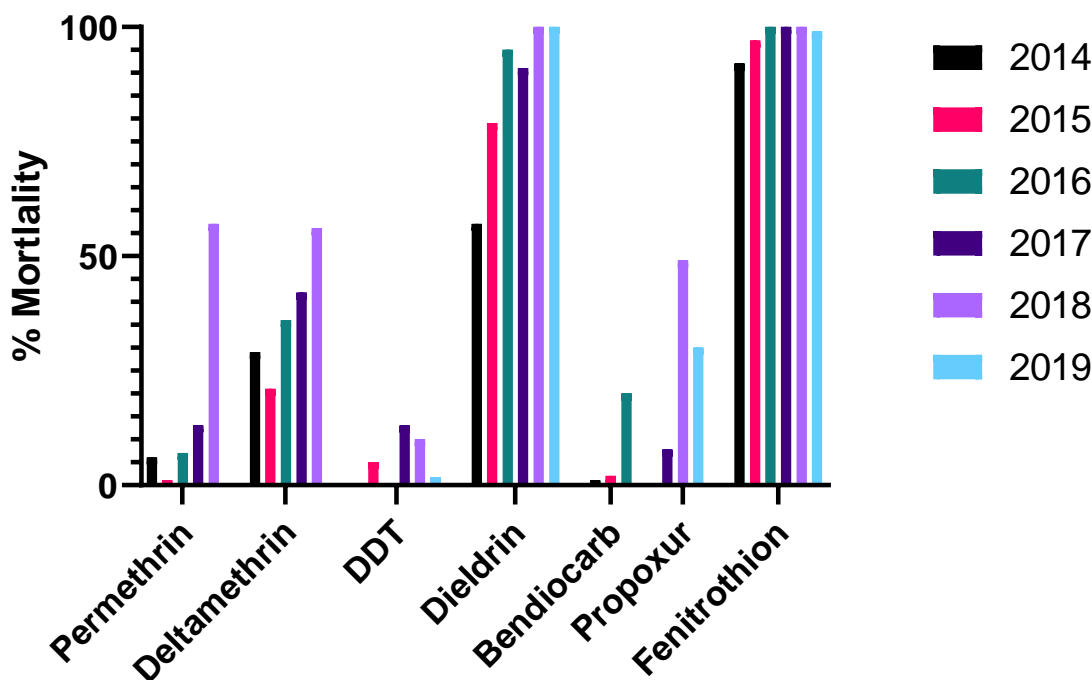

Supplement: Supplementary file 5 — Additional file 5: Figure S4. Aedes aegypti colony profiling. Mortality rates 24 hours after exposure for 2 strains of Ae. aegypti. [file 13071_2019_3774_MOESM5_ESM.pdf]
