# Supplementary material for: Characterisation of Anopheles strains used for laboratory screening of new vector control products
Source: Parasit Vectors. 2019 Nov 5;12:522. doi: 10.1186/s13071-019-3774-3 (PMC6833243; doi:10.1186/s13071-019-3774-3)
Supplement: Supplementary file 6 — Additional file 6: Table S2. Topical and tarsal resistance ratios of additional insecticides. Abbreviation: ND, not done. [file 13071_2019_3774_MOESM6_ESM.pdf]

Additional file 6: Table S2. Topical and tarsal resistance ratio (relative to Kisumu) of additional insecticides. ND = not done.

Resistant Ratio	FUMOZ-R	Tiassalé 13
Bendiocarb		
Topical	2.25	1.26
95% CI	(2.21 – 6.33)	(1.19 – 4.43)
Tarsal	3.21	11.7
95% CI	(2.53 – 4.08)	(7.83 – 17.57)
DDT		
Topical	0.83	ND
95% CI	(0.56 – 3.40)	ND
Pirimiphos-Methyl		
Topical	ND	2.68
95% CI	ND	(1.63 – 6.67)
Tarsal	1.32	1.61
95% CI	(1.10 – 1.59)	(1.30 – 2.00)
