# Supplementary material for: Characterisation of Anopheles strains used for laboratory screening of new vector control products
Source: Parasit Vectors. 2019 Nov 5;12:522. doi: 10.1186/s13071-019-3774-3 (PMC6833243; doi:10.1186/s13071-019-3774-3)
Supplement: Supplementary file 7 — Additional file 7: Table S3. Additional genotype (%) and allele frequencies for extra-diagnostic SNPs. [file 13071_2019_3774_MOESM7_ESM.pdf]

Additional file 7: Table S3. Additional genotype (%) and allele frequencies for extra diagnostic SNPs.

Strain	Genotype (%)				Date of last screening
**2LA Inversion**	2L+a (wild type)	2La/2L+a (hetero)	2La (inversion)	**Allele frequency**	
Kisumu	48	35	17	0.6	Apr 2013
Tiassalé 13	45	11	44	0.7	Mar 2013
Moz	100	0	0	0	Jul 2013
**GSTe2 114**	II (wild type)	IT (hetero)	TT (mutant)	**Allele frequency**	
Kisumu	15	64	21	0.5	Apr 2013
Tiassalé 13	73	27	0	0.1	Mar 2013
FUMOZ-R	100	0	0	0	Feb 2014
